# Expression profiling and bioinformatics analysis of circulating microRNAs in patients with acute myocardial infarction

**DOI:** 10.1002/jcla.23099

**Published:** 2019-11-12

**Authors:** Zhixiong Zhong, Heming Wu, Wei Zhong, Qifeng Zhang, Zhikang Yu

**Affiliations:** ^1^ Center for Cardiovascular Diseases Meizhou People's Hospital (Huangtang Hospital) Meizhou Academy of Medical Sciences Meizhou Hospital Affiliated to Sun Yat‐sen University Meizhou China; ^2^ Guangdong Provincial Key Laboratory of Precision Medicine and Clinical Translational Research of Hakka Population Meizhou People's Hospital (Huangtang Hospital) Meizhou Academy of Medical Sciences Meizhou Hospital Affiliated to Sun Yat‐sen University Meizhou China; ^3^ Center for Precision Medicine Meizhou People's Hospital (Huangtang Hospital) Meizhou Academy of Medical Sciences Meizhou Hospital Affiliated to Sun Yat‐sen University Meizhou China; ^4^ Guangdong Provincial Engineering and Technological Research Center for Molecular Diagnostics of Cardiovascular Diseases Meizhou China; ^5^ Meizhou Municipal Engineering and Technological Research Center for Molecular Diagnostics of Cardiovascular Diseases Meizhou China; ^6^ Meizhou Municipal Engineering and Technological Research Center for Molecular Diagnostics of Major Genetic Disorders Meizhou China

**Keywords:** acute myocardial infarction, biomarker, expression profiles, microRNA, RNA sequencing

## Abstract

**Objective:**

Acute Myocardial Infarction (AMI) is the most severe type of coronary atherosclerotic heart diseases. MiRNA is a class of endogenous noncoding small molecule RNA, which plays an important regulatory role in the development of some diseases.

**Methods:**

We examined the miRNA expression profiles in 16 patients with AMI compared with 6 non‐AMI controls using RNA sequencing.

**Results:**

Compared with the miRNA expression profiles of non‐AMI controls, a total of 181 differentially expressed miRNAs were discriminated in AMI patients, of which 96 upregulated miRNAs and 85 downregulated miRNAs. The top ten upregulated miRNAs were as follows: miR‐449a‐5p, miR‐126‐5p, miR‐93‐5p, miR‐199a‐3p, miR‐4454, miR‐6880‐3p, miR‐3135a, miR‐548ad‐5p, miR‐4508, and miR‐556‐5p; while the top ten downregulated were as follows: miR‐6805‐5p, miR‐1228‐5p, miR‐939‐5p, miR‐615‐3p, miR‐6780a‐5p, miR‐6857‐3p, miR‐5088‐55p, miR‐7155‐3p, miR‐184, and miR‐4525. And the qRT‐PCR results of differentially expressed miRNAs showed the same result as high‐throughput sequencing data. For these 181 differentially expressed miRNAs, 19 841 target genes were predicted by GO analysis. The enrichment analysis revealed 2061 involved in biological processes, 353 in molecular function and 303 in cellular components. To identify biological pathways in AMI as compared to non‐AMI, the target genes of differentially expressed miRNAs were mapped to the classical signal transduction pathway in KEGG, indicating that 214 classes were enriched. ROC analysis showed that the circulating miRNAs had the important value for AMI diagnosis and supported the previous conclusions that circulating miRNAs were effective to diagnose the AMI as a novel biomarker.

**Conclusions:**

Our findings require further research to confirm. It may provide a meaningful reference for the diagnosis and treatment of AMI.

## 1 INTRODUCTION

Acute coronary syndrome (ACS) is a group of clinical syndromes characterized by rupture or invasion of coronary atherosclerotic plaques secondary to complete or incomplete occlusive thrombosis, including ST‐segment elevation myocardial infarction (STEMI), non‐ST‐segment elevation myocardial infarction (NSTEMI), and unstable angina (UA). STEMI and NSTEMI are collectively referred to as acute myocardial infarction (AMI).^1^, ^2^

Acute myocardial infarction is the most serious type of coronary atherosclerotic heart diseases. It has the characteristics of rapid onset, rapid progression, and high mortality. The disease is the primary cause of the death of non‐tumor diseases in developed countries. In recent year, the incidence of AMI in some countries has also increased year by year and has a trend of youth. At present, the pathogenesis of cardiovascular disease has not yet been fully elucidated. Inflammatory factors, protease molecules, and abnormal expression of apoptotic molecules are related to the injury of cardiac muscle cells and cardiovascular diseases.^3^

Timely, correct diagnosis and early reperfusion treatment can significantly reduce the mortality of AMI. At present, the biomarkers for diagnosis of AMI include creatine kinase isoenzyme (CK‐MB) and troponin cTnI (cTnI). CTnI is considered the gold standard for the diagnosis of AMI.^4^, ^5^ However, studies showed that cTnI increased significantly during the treatment of various diseases,^6^, ^7^ and there were defects in the early diagnosis of AMI and myocardial infarction. However, the specificity of CK‐MB in diagnosing AMI was less than cTnI, which has been gradually replaced by cTnI. Therefore, it is of great significance to find more sensitive and specific, more targeted AMI diagnostic markers in time windows.^8^ Studies have found that microRNAs (miRNAs) play an important role in disease progression and they can be used as biomarkers. Accumulated evidence reveals miRNAs as a biomarker of AMI and its associated symptoms, including vulnerable atherosclerotic plaques, and their role in disease diagnosis, platelet activation monitoring, and prognostic outcome prediction.^8^

MiRNA is a class of single strand non‐coded RNA with length 18‐22 bp, which regulates the expression of various genes in the body. They are stable in peripheral blood and thus have potential in diagnosing related diseases.^9^ MiRNAs are released into biological fluids, including blood, from dying cells such as necrotic cells, or actively secreted from living cells under‐stimulation. The stability of mature miRNAs is controlled by cis‐acting modifications, protein complex formation and exposure to nucleases. A simple code that dictates miRNA half‐life may not exist.^10^, ^11^ The analysis of the expression of miRNA in tissue or cell samples provides important information for the study of the biological functions of these molecules. The expression profiles of circulating miRNA in healthy people and patients with various diseases are significantly different.^9^ In recent years, related studies have confirmed that miRNAs can regulate the function of cardiac myocytes, and the abnormal miRNAs content may lead to the occurrence of cardiovascular disease.^12^, ^13^, ^14^, ^15^, ^16^

We conducted a hospital‐based case‐control study. In this study, we applied high‐throughput sequencing technology to detect the miRNA in all subjects. Studies of the differential expression of miRNA in patients with AMI, the role of miRNA in AMI and the connection between miRNA and the occurrence and trend of AMI may provide new ideas for the early diagnosis and the clinical risk stratification of AMI.

## 2 MATERIAL AND METHODS

### 2.1 Subjects

All the subjects in this study visited Department of Cardiovascular Diseases of Meizhou People's Hospital for reasons of cardiovascular meditation or examination. Inclusion criteria: cardiovascular risk factors, symptoms of chest pain, ischemic changes in ECG, or elevated myocardial enzymes. Exclusion criteria: impaired left ventricular ejection fraction ≤45%, congestive heart failure, chronic kidney or hepatic disease, AMI, and malignant disease. All subjects in this study were examined by laboratory tests, electrocardiograph (ECG), and coronary angiography. Acute myocardial infarction was diagnosed by coronary angiography, dynamic evolution of electrocardiogram, and dynamic changes of serum markers. The patients with ST‐segment elevation were diagnosed as STEMI, and those with no ST‐segment elevation were diagnosed as NSTEMI. Twenty‐two subjects including 12 males and 10 females (1.2:1) involved in this study. Twenty‐two subjects were selected for the present study and were classified into 2 groups: non‐AMI and AMI group, which visited Meizhou People's Hospital (Huangtang Hospital), Meizhou Hospital Affiliated to Sun Yat‐sen University, Guangdong province of China through February 2016 to April 2017, which age from 38 to 73 years. The study was conducted on the basis of the Declaration of Helsinki and was supported by the Ethics Committee of the Meizhou People's Hospital (Huangtang Hospital).

### 2.2 Samples collection and total RNA extraction

Plasma samples were collected before the coronary angiography surgery and were immediately frozen at −80°C for RNA‐Seq or qRT‐PCR analyses. Two samples were taken at the time of admission for AMI patients, one for the extraction of whole blood RNA and one for the detection of CK‐MB and cTnI.

Whole blood samples (6 mL from peripheral venous blood) were collected from patients with AMI at the onset of symptoms and non‐AMI controls. Blood samples were taken from an antecubital vein using EDTA anticoagulant tubes, on the upside‐down gently mix after 10 times, immediately saved the blood of 4°C, separated plasma in 1 hour and were centrifuged at 800 *g* centrifugal 10min get the upper plasma samples, transferred the plasma to 1.5 mL RNA (RNase‐free) centrifuge tube for extraction of RNA, packed stored at −80°C.

Total RNA was extracted from the plasma using TRIzol reagent (Invitrogen) according to the manufacturer's instructions. The quantity and purity of total RNA were evaluated by Nanodrop 2000 and used the RNA Nano 6000 Assay Kit of the Agilent Bioanalyzer 2100 system (Agilent Technologies) to analyze RNA integrity.

### 2.3 Libraries construction and inspection

Library construction of small RNA used by TruSeq Small RNA Prep Kit, the use of 3′ and 5′ end special structure (5′ end phosphate group, complete 3′ end hydroxyl) with total RNA as the starting sample, directly to the small RNA, at both ends with joints, and then reversed transcriptase to synthesize cDNA. After PCR amplification, the target DNA fragment was separated by PAGE gel electrophoresis, and the cDNA library was recovered by cutting glue.

After the libraries were constructed, used Qubit2.0 preliminary quantitative and dilution library to 1 ng/μL, then the insert size of libraries were detected by Agilent 2100. The insert size in line with expectations, accurate quantification was carried out used the Q‐PCR method on the effective concentration of Library (Library of effective concentration >2 nmol/L), in order to guarantee the quality of the library.

### 2.4 High‐throughput sequencing

Sequencing libraries were generated using NEBNext^®^ Multiplex Small RNA Library Prep Set for Illumina^®^ (NEB) according to the manufacturer's protocol. After the qualification of the library, the different libraries were sequenced in accordance with the requirement of the effective concentration and the requirement of the amount of data of the machine under the target pooling, and then library sequencing was carried out on Illumina HiSeq 2500 platform according to the commercially available protocols in ShenZhen Realomics Inc.

### 2.5 Identification of differently expressed genes

Differential expression analysis of two groups samples was performed using the DEGseq (2010) R package. *P*‐value was adjusted using *q*‐value. And* q*‐value <.05 and |log2(foldchange)| > 1 was set as the threshold for significantly differential expression by default.

### 2.6 Quantitative real‐time polymerase chain reaction (qRT‐PCR)

To validate the reliability of RNA sequencing data, differentially expressed miRNAs were randomly selected and qRT‐PCR was performed to examine the expression level of miRNAs. Total RNA was extracted from the plasma using TRIzol reagent (Invitrogen) according to the manufacturer's instructions. Extracted RNA was converted to cDNA, and qRT‐PCR reactions were performed using Luna Universal One‐Step RT‐qPCR kits (New England Biolabs). We verified the expression of these miRNAs from PBMCs of AMI patients and healthy controls with qRT‐PCR using glyceraldehyde 3‐phosphate dehydrogenase (GAPDH) as the reference gene with the 2^−ΔΔCT^ method.

### 2.7 GO and KEGG enrichment analysis

The miRNA target genes were classified according to the principle of classification by Gene Ontology (http://www.geneontology.org/). GO gathers information from Gene Ontology and the NCBI database and annotates and classifies genes according to the biology process, molecular function, and cellular location. KEGG (http://www.genome.jp/kegg/) is a comprehensive database for systematic analysis of gene function. It is based on the related knowledge of hand‐painted metabolic pathways, divided into categories: metabolism, genetic information processing, cellular processes, environmental information processing, organismal systems, and human diseases. Each category is divided into some sub‐items.

### 2.8 Statistical analysis

SPSS statistical software version 19.0 was used for data analysis. Data were reported as the means ± SD. Chi‐square and ANOVA tests were used to analyze the differences between the two groups. Statistical significance was set at a *P* < .05. The receiver operating characteristic (ROC) curve was performed and the area under the ROC curve (AUC) was calculated to evaluate the diagnostic value of some differentially expressed miRNAs were randomly selected for differentiating between AMI patients and non‐AMI controls.

## 3 RESULTS

### 3.1 The subjects' clinical characteristics

A total of 16 AMI patients and 6 non‐AMI controls were recruited in the study. The clinical characteristics of the 22 subjects in this study were presented in Table 1. There were higher systolic BP, TC, and LDL‐C in the AMI patients than non‐AMI controls (*P* = .003, .021, and .016, respectively). There were no statistical differences in age, sex, smoking, drinking, diastolic BP, hypertension, diabetes, hyperlipidemia, triglycerides, and HDL‐C between the AMI patients and the non‐AMI controls.

**Table 1:** The baseline clinical characteristics

Variable	Non‐AMI	AMI	*P‐*value
Age (y)	53.50 ± 8.67	55.38 ± 10.28	.321
Sex (male)	3 (50%)	9 (56.25%)	.560
Smoking	3 (50%)	11 (68.75%)	.392
Drinking	2 (33.33%)	6 (37.5%)	.615
Systolic BP (mm Hg)	129.5 ± 7.26	136.2 ± 22.7	.003
Diastolic BP(mm Hg)	82.67 ± 9.52	83.00 ± 16.77	.208
Hypertension	1 (16.7%)	8 (50%)	.145
Diabetes	1 (16.7%)	3 (18.75%)	.698
Hyperlipidemia	1 (16.7%)	6 (37.5%)	.314
TC, mmol/L	4.40 ± 1.09	6.31 ± 0.59	.021
TG, mmol/L	1.18 ± 0.54	1.91 ± 1.53	.052
HDL‐C, mmol/L	1.06 ± 0.48	1.22 ± 0.31	.160
LDL‐C, mmol/L	2.24 ± 0.17	2.99 ± 1.09	.016

Abbreviations: HDL‐C, high‐density lipoprotein cholesterol; LDL‐C, low‐density lipoprotein cholesterol; TC, total cholesterol; TG, triglycerides.

### 3.2 Differentially miRNA expressed profiles in AMI

**Figure 1:**
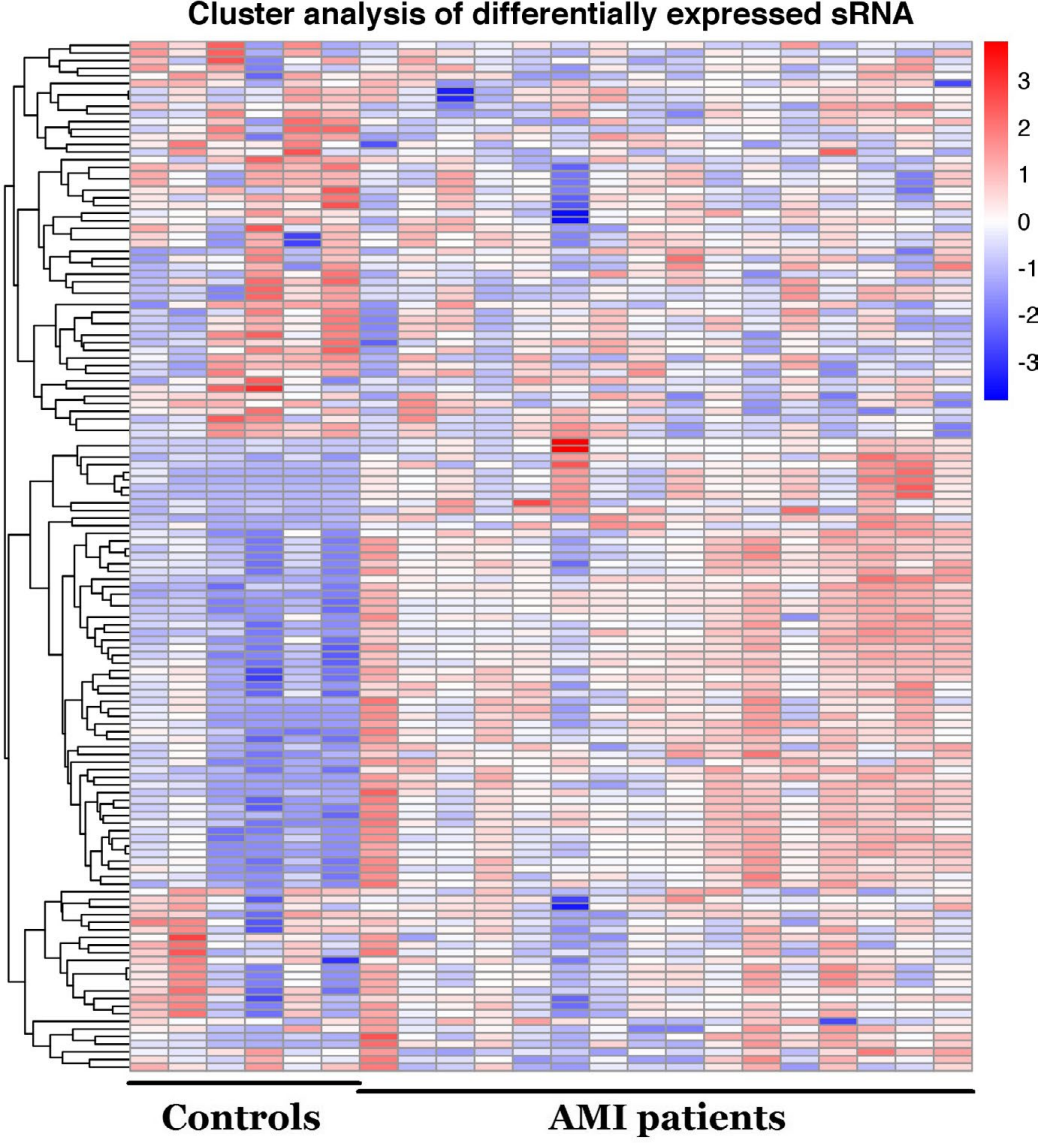
Hierarchical clustering of miRNAs in patients with or without AMI

MiRNAs were analyzed with strict data quality control, and a total of 2135 miRNAs were found. The miRNA expressed profiles between the patients with AMI and the non‐AMI controls was shown in Figure 1. The green and red in the picture indicate that the relative expression is reduced and raised, respectively.

**Figure 2:**
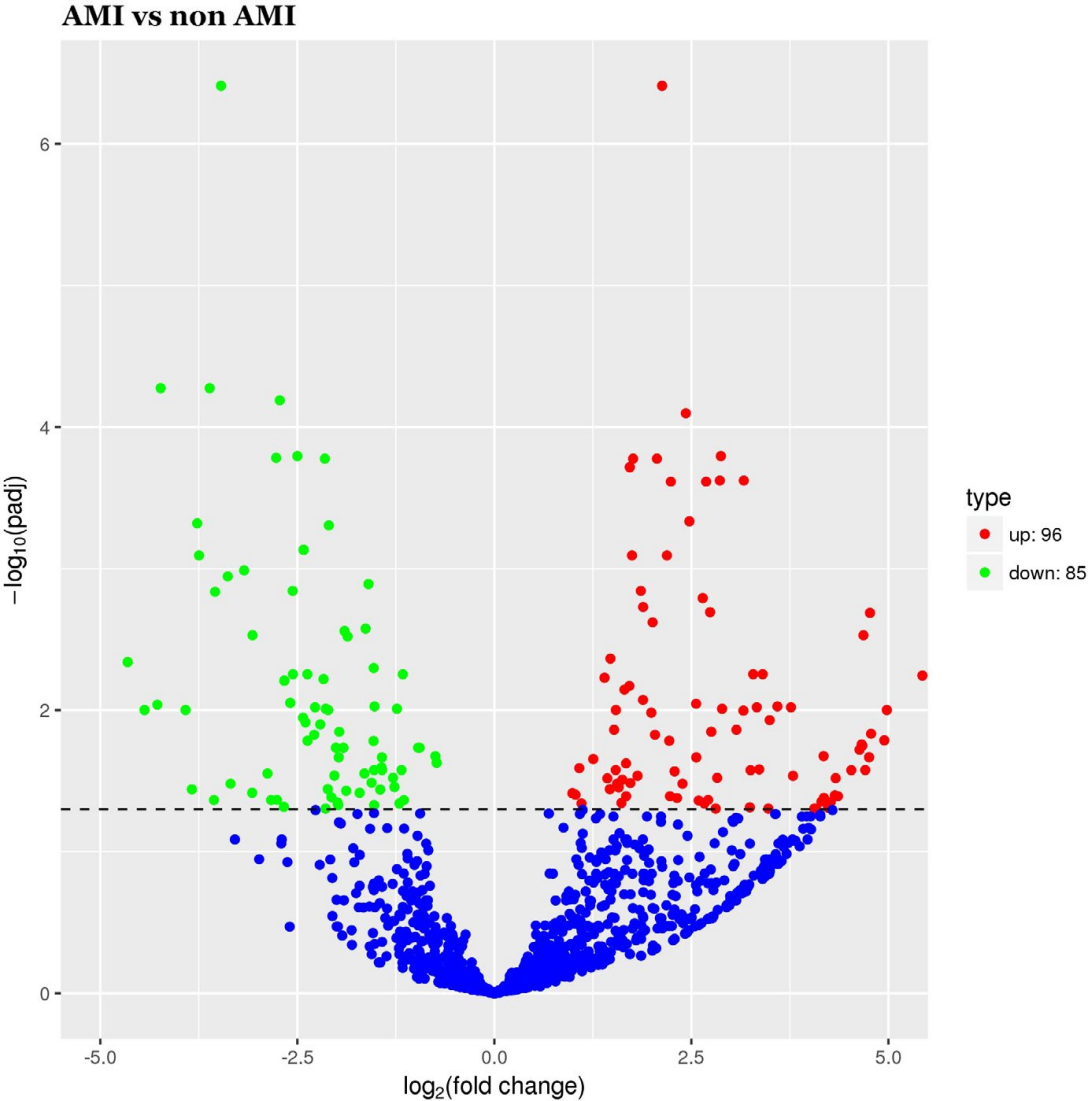
Volcano plot of differential miRNA expression. *X*‐axis: log_2_(fold change); *Y*‐axis: −1 × log_10_ (corrected *q*‐value) for each probes

MiRNAs were considered significantly upregulated if relative expression levels showed a fold change (FC) ≥ 1 and *P* ≤ .05, and considered significantly downregulated with FC ≤ −1 and *P* ≤ .05. As the volcano plot is shown in Figure 2, compared with the miRNA expression profiles of non‐AMI controls, a total of 181 differentially expressed miRNAs were discriminated in AMI patients, of which 96 upregulated miRNAs and 85 downregulated miRNAs.

The top ten upregulated miRNAs were as follows: miR‐449a‐5p, miR‐126‐5p, miR‐93‐5p, miR‐199a‐3p, miR‐4454, miR‐6880‐3p, miR‐3135a, miR‐548ad‐5p, miR‐4508, and miR‐556‐5p; while the top ten downregulated were as follows: miR‐6805‐5p, miR‐1228‐5p, miR‐939‐5p, miR‐615‐3p, miR‐6780a‐5p, miR‐6857‐3p, miR‐5088‐55p, miR‐7155‐3p, miR‐184, and miR‐4525.

### 3.3 Target prediction and functional analysis of differentially expressed miRNAs

**Figure 3:**
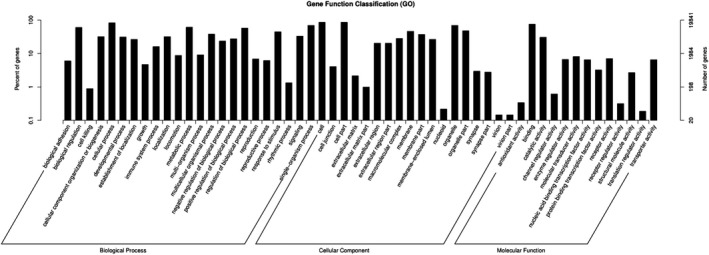
GO analysis of differentially expressed miRNAs that covers three domains: biological process, cellular component, and molecular function. *X*‐axis: GO terms of biological process, cellular component, and molecular function. The green column indicates the biological process, the red column indicates cellular component, and the blue column indicates molecular function. *Y*‐axis on the left: numbers of genes (miRNAs)

For these 181 differentially expressed miRNAs, 19 841 target genes were predicted by GO analysis. Functional analysis revealed that 2061 GO terms found to be involved in biological processes, 353 in molecular function, and 303 in cellular components were significantly enriched (*P* < .05). The most common GO categories were cell part, organelle, biological regulation, metabolic process, cellular process, membrane, regulation of biological process, membrane‐enclosed lumen, localization, multicellular organismal process, developmental process, binding, cellular component organization or biogenesis, single‐organism process, response to stimulus, extracellular region part, positive regulation of biological process, establishment of localization, catalytic activity. The results as shown in Figure 3.

### 3.4 KEGG pathway analysis of targets of differentially expressed miRNAs

**Figure 4:**
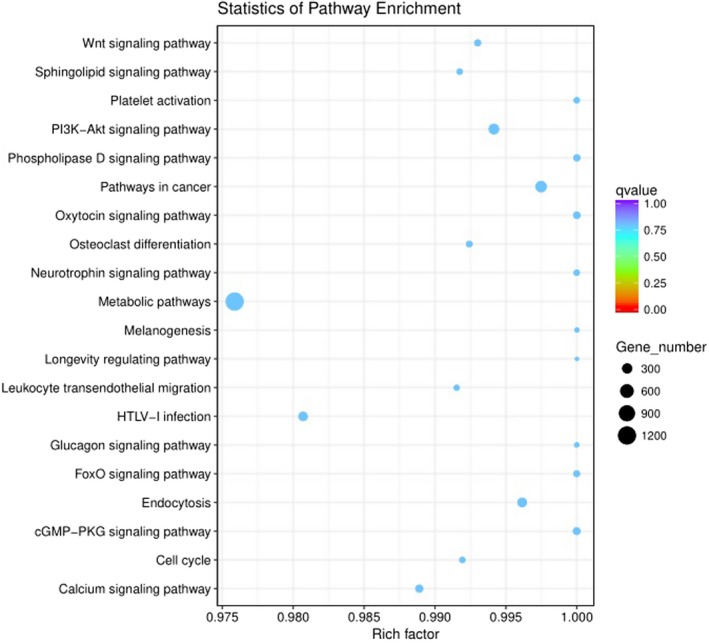
KEGG Pathway analysis of differentially expressed miRNAs. Pathway analysis is a functional analysis mapping genes to KEGG pathway and other pathway databases. The lower the *P*‐value, the more significant the pathway

To identify the biological pathways in AMI compared with non‐AMI, 214 categories were enriched by mapping the target genes of differentially expressed miRNAs into classical signaling pathways in the KEGG. The top 20 pathways according to *P*‐value showed in Figure 4.

### 3.5 qRT‐PCR validation of miRNA expression

**Figure 5:**
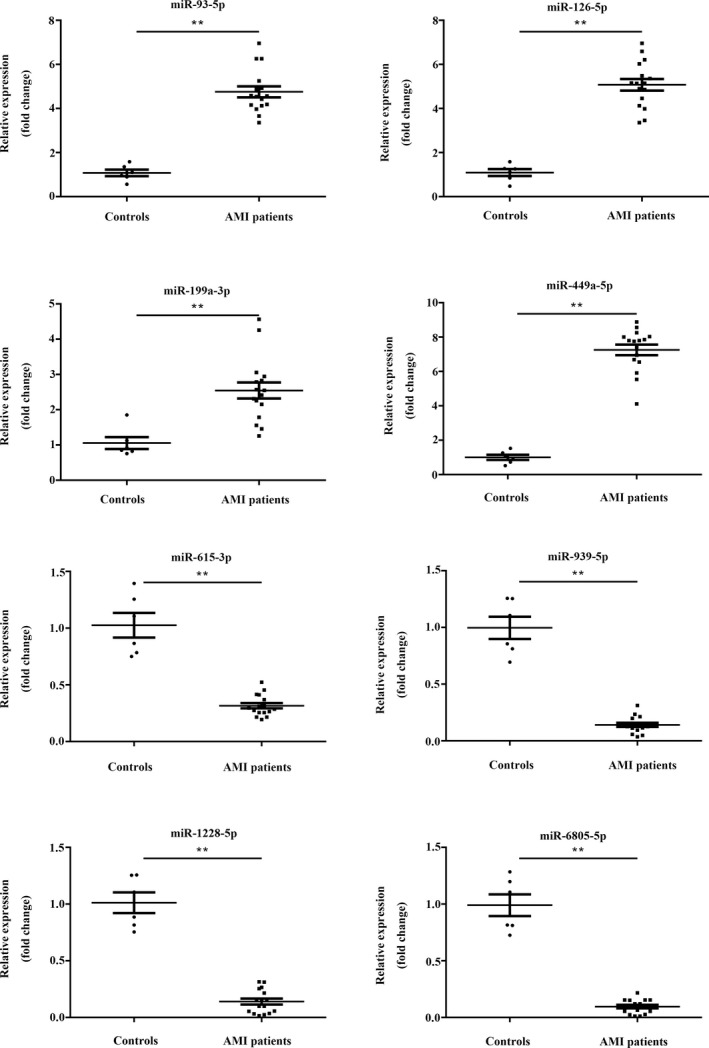
Confirmation of differentially expressed in microRNAs by qRT‐PCR. **P < .01

To validate differential expression profiles of miRNA by high‐throughput sequencing, four upregulated miRNAs (miR‐93‐5p, miR‐126‐5p, miR‐199a‐5p, and miR‐499a‐5p) and four downregulated miRNAs (miR‐615‐3p, miR‐939‐5p, miR‐1228‐5p, and miR‐6805‐5p) were randomly selected. The qRT‐PCR results of differentially expressed miRNAs showed the same result as high‐throughput sequencing data. The results as shown in Table 2 and Figure 5.

**Table 2:** Confirmation of differentially expressed microRNAs by qRT‐PCR

microRNA	qRT‐PCR						Fold change by high‐throughput sequencing
	Average Ct in AMI	GAPDH average Ct in AMI	AMI ΔCt (Average Ct − GAPDH Average Ct)	Non‐AMI ΔCt (Average Ct − GAPDH Average Ct)	ΔΔCt (AMI ΔCt − non‐AMI ΔCt)	Fold change	
hsa‐miR‐93‐5p	29.92	23.22	6.70	8.88	−2.18	4.5315	4.0225
hsa‐miR‐126‐5p	31.21	22.90	8.31	10.67	−2.36	5.1337	4.3720
hsa‐miR‐199a‐5p	31.11	23.74	7.37	8.73	−1.36	2.5668	2.1108
hsa‐miR‐499a‐5p	31.68	22.38	9.30	12.19	−2.89	7.4127	6.7379
hsa‐miR‐615‐3p	31.06	23.60	7.46	5.70	1.76	0.2952	0.2385
hsa‐miR‐939‐5p	30.43	23.46	6.97	4.12	2.85	0.1387	0.1768
hsa‐miR‐1228‐5p	27.68	22.21	5.47	2.14	3.33	0.0994	0.1192
hsa‐miR‐6805‐5p	29.37	23.79	5.58	2.32	3.26	0.1044	0.0818

### 3.6 ROC analysis

**Figure 6:**
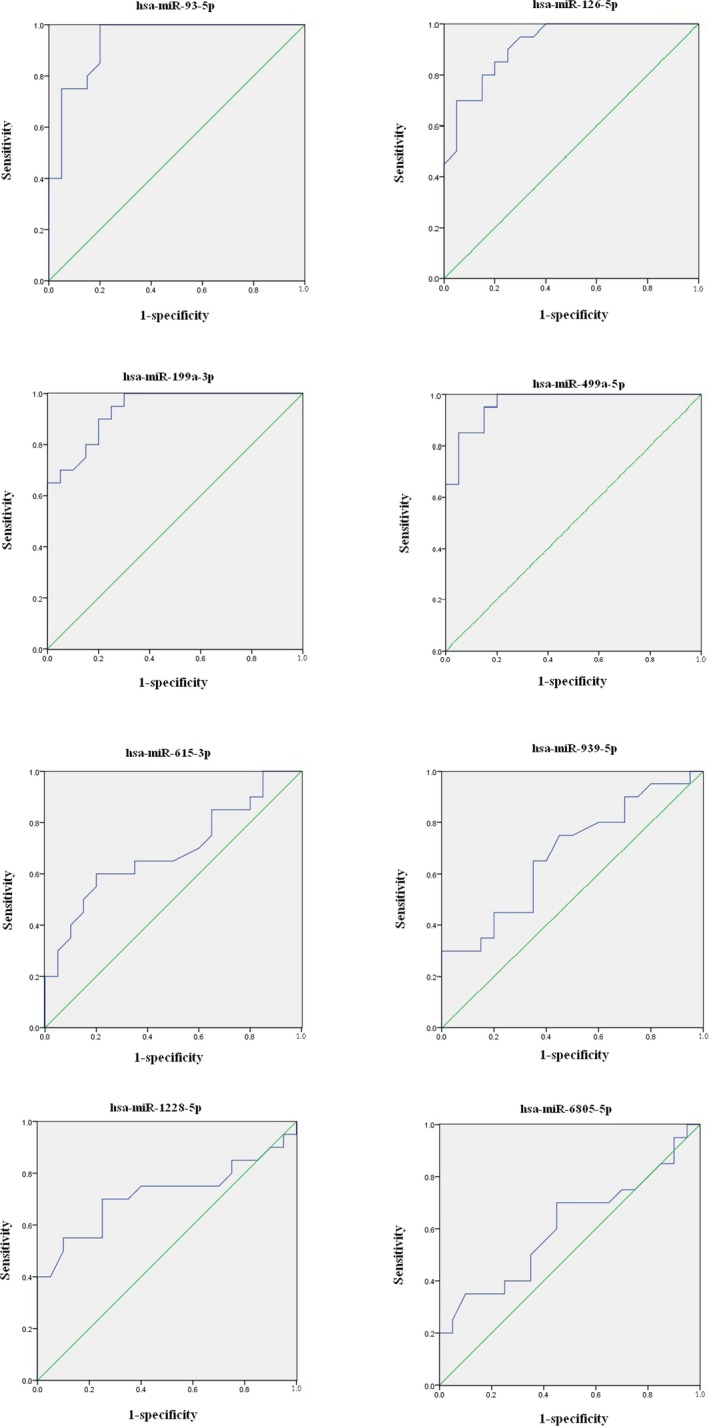
ROC curve analyzed the diagnostic value of circulating differentially expressed miRNAs were randomly selected for differentiating between AMI patients and non‐AMI controls

ROC analysis was calculated to evaluate the diagnostic value of some differentially expressed miRNAs were randomly selected for differentiating between AMI patients and non‐AMI controls. MiRNAs with a *P* < .05 and an AUC > 0.6 were selected as successful distinguishing markers between AMI patients and non‐AMI controls (Figure 6). More specifically, the ROC curves yielded the following AUCs: miR‐93‐5p (AUC = 0.936, upregulated), miR‐126‐5p (AUC = 0.919, upregulated), miR‐199a‐5p (AUC = 0.936, upregulated), miR‐499a‐5p (AUC = 0.965, upregulated), miR‐615‐3p (AUC = 0.688, downregulated), and miR‐1228‐5p (AUC = 0.716, downregulated) were found to discriminate AMI patients from non‐AMI controls, showed in Table 3.

**Table 3:** Areas under the ROC curve and predictive value of four upregulated miRNAs and four downregulated miRNAs

	hsa‐miR‐93‐5p	hsa‐miR‐126‐5p	hsa‐miR‐199a‐5p	hsa‐miR‐499a‐5p	hsa‐miR‐615‐3p	hsa‐miR‐939‐5p	hsa‐miR‐1228‐5p	hsa‐miR‐6805‐5p
2^−ΔΔCt^	1.77 ± 0.51	1.74 ± 0.40	1.73 ± 0.45	2.13 ± 0.56	0.69 ± 0.29	0.63 ± 0.25	0.73 ± 0.25	0.80 ± 0.23
95% CI	0.862‐1.000	0.838‐1.000	0.868‐1.000	0.917‐1.000	0.520‐0.855	0.509‐0.843	0.547‐0.885	0.917‐1.000
*P*‐value	<.001	<.001	<.001	<.001	.042	.057	.019	.250
AUC	0.936	0.919	0.936	0.965	0.688	0.676	0.716	0.606
Cut‐off point	1.38	1.39	1.30	1.61	0.70	–	0.815	–
Specificity (%)	95	85	85	95	80	–	70	–
Sensitivity (%)	75	80	80	85	60	–	70	–

Note

2^−ΔΔCt^, relative expression of miRNAs (mean ± SD); 95% Cl, 95% confidence interval; AUC, area under the ROC curve.

## 4 DISCUSSION

The ideal AMI biomarkers should meet three requirements^17^: (a) ease of acquisition: samples must be obtained with a minimum invasive method and are more easily obtained, such as plasma, serum, and urine; (b) predictability: biomarkers should have a relatively long half‐life in the blood to ensure the predictability of detection. The expression level of biomarkers is closely related to the degree of healing of the myocardial injury; and (c) reliability: the detection method should be fast, accurate, sensitive, and cheap. There is no special requirement for the instrument and equipment, and it is easy to detect. Most of the biological markers used clinically are proteins and peptides.^18^ New biomarkers, such as molecular and genetic levels, are still being studied.^19^ In recent years, miRNA has become a new type of biomarker because of its organization or cell specificity and its involvement in a variety of pathological and physiological processes.^20^, ^21^, ^22^

The previous main research results of the relationship between miRNA and AMI were summarized. Studies found that compared with non‐AMI controls, the serum miR‐1 level in AMI patients increased significantly and returned to normal level after 2 weeks with drug treatment.^23^ In the model of myocardial infarction in mice with coronary artery ligature, the serum level of miR‐1 in mice increased rapidly after the onset of AMI.^24^ Compared with the normal control group, the serum miR‐133 level in AMI patients increased by 4.4 times and returned to normal level after 7 days. The researchers also proved that serum miR‐133 was associated with cTnT, but the miR‐133 peak appeared earlier than cTnT.^22^ The levels of serum miR‐133a and miR‐133b were independently associated with the increase of troponin, and the level of miR‐133a was also associated with the risk of death in AMI patients.^25^ The study of miRNA in cardiac myocytes of AMI patients showed that miR‐208 was not expressed in normal myocardium. It was found that miR‐208 had higher cardiac specificity than miR‐1, miR‐133, and miR‐499. In addition, in the AMI rat model, miR‐208 was highly expressed at 1 hour after coronary artery ligation, and the peak appeared in 3 hours and began to decrease gradually in 6‐12 hours, disappeared after 24 hours.^26^ Similar to miR‐208, miR‐499 was not detected in healthy human serum but increased in serum of AMI patients, which hardly detected in congestive heart failure and normal heart.^27^ By studying 92 patients with NSTEMI, the researchers found that the serum miR‐499‐5p in NSTEMI patients was significantly increased about 80 times compared with the healthy control group. So miR‐499 can be used as a biomarker for the diagnosis of AMI.^28^

According to the KEGG pathway database, the signal pathways associated with cardiovascular disease in our findings include the cGMP‐PKG signaling pathway and Oxytocin signaling pathway. cGMP is a second messenger that is produced by guanylate cyclase (GC) acting on guanosine triphosphate (GTP), which acts through receptor‐protein interactions within the cell. As an important intracellular messenger, cGMP has three main targets: cGMP‐dependent protein kinase or protein kinase G (PKG), cGMP‐regulated phosphodiesterases (PDE), and cGMP‐gated cation channels. Cyclic phosphate guanylate‐dependent protein kinase (PKG), a silk/threonine‐protein kinase widely found in eukaryotic cells, is considered to be the most important downstream target of cGMP. In cardiomyocytes, PKG directly phosphorylates members of the TRPC6, a typical family of transient potential receptors, inhibits Ca^2+^ conduction in this non‐selective ion channel, G‐alpha‐q agonist‐induced NFAT activation, and myocyte hypertrophy. PKG also opens the mitochondrial ATP‐sensitive K^+^ channel, followed by ROS release to trigger cardioprotection.^29^, ^30^, ^31^ Oxytocin (OT) is a cyclic polypeptide consisting of 9 amino acid residues. The disulfide bond of cysteine at the 1st and the 6th position forms a ring with a tripeptide tail molecular. Oxytocin belongs to the transmembrane receptor of the G protein‐coupled receptor superfamily. The major signaling pathway is the Gq/PLC/Ins3 pathway, which exerts a physiological effect by binding to its receptor. Studies have shown that in the cardiovascular system, OTR is associated with the ANP‐cGMP and NO‐cGMP pathways, reducing contractility and contractility and increasing vasodilation.^32^, ^33^ Our findings showed that modulated miRNAs may regulate the functions of target genes in these signaling pathways during the formation and development of AMI.

At last, the ROC curve of four upregulated miRNAs and four downregulated miRNAs was plotted to investigate the informativeness for AMI diagnosis. We acquired an AUC of 0.936, 0.919, 0.936, and 0.965 in four upregulated miRNAs, an AUC of 0.688 and 0.716 in two downregulated miRNAs, individually. By using denoted threshold score of circulating miR‐93‐5p, miR‐126‐5p, miR‐199a‐5p, and miR‐499a‐5p, the high specificity and sensitivity were achieved for detection of AMI from non‐AMI control group. So, the data showed that the circulating miRNAs had the important value for AMI diagnosis and supported the previous conclusions that circulating miRNAs were effective to diagnose the AMI as novel biomarkers.^34^, ^35^, ^36^ Next step, we will carry out ROC analysis of the remaining 92 upregulated miRNAs and 81 downregulated miRNAs, in order to find more candidate miRNAs with diagnostic value.

So far, cTnT is still the gold standard for clinical diagnosis of AMI but has certain limitations. cTnT begins to rise within 3‐4 hours after the onset of myocardial injury, eliminating early AMI diagnosis within first 1‐2 hours. It is mainly excreted by the kidneys in patients with end‐stage renal disease was troponin elevation, renal function will, therefore, affect the sensitivity of troponin testing to a certain extent, thus affecting the specificity of troponin testing.^37^, ^38^ Owing to the significant roles of miRNA in disease development, they can be used as biomarkers. A study has revealed an important role of miRNAs as biomarkers of AMI and its associated symptoms, and their role in disease diagnosis, platelet activation monitoring, and prognostic outcome prediction.^11^ And study identified circulating monocytes as novel biomarkers and carriers for the cell‐specific transfer of miRNAs in the early stage of myocardial infarction.^39^ Some findings strengthen the potential of applying miRNAs, such as miR‐708, to reconstitute lost cardiomyocytes in injured hearts and may provide a novel candidate for promoting heart regeneration.^40^ Therefore, with further improving the miRNA detection technology, miRNA may become a marker for early diagnosis and treatment of AMI.

More and more miRNAs have been discovered, and the biological function has been gradually clarified. However, it is still a major problem in the field of miRNA research to find out what functions miRNA has, including the target genes of miRNA, the results of the interaction between miRNA and the mechanism of miRNA. The research of miRNA is still in its start stage. Some researchers anticipate that future studies in miRNA will change the way we understand how cells communicate, which will contribute to the way we treat some diseases.^41^ Some experts believe that investigating the roles of miRNAs in the setting of AMI has provided new insights into the development of disease underlying acute myocardial ischemia/reperfusion injury, and has discovered novel biomarkers and therapeutic targets for detecting and treating AMI. And it may have the therapeutic potential to improve clinical outcomes in AMI patients.^42^ It will bring new hope for AMI patients.

In this study, a relatively small number of samples were enrolled to study differential expression of miRNAs in patients with AMI from non‐AMI in order to find a novel biomarker for AMI diagnosis. A larger number of clinical samples studies should be required to support our results. However, luckily and importantly, we gained some candidate miRNAs as novel biomarker for AMI diagnosis.

In summary, our findings suggested that it has significant changes in the expression of multiple miRNAs in AMI compared with non‐AMI. In the course of the formation and development of AMI, the differential expression of miRNA may be related to the cGMP‐PKG signaling pathway and Oxytocin signaling pathway. ROC analysis showed that the circulating miRNAs had the important value for AMI diagnosis and supported the previous conclusions that circulating miRNAs were effective to diagnose the AMI as a novel biomarker. Our findings require further research to confirm. It may provide a meaningful reference for the diagnosis and treatment of AMI.

## CONFLICTS OF INTEREST

The authors declare no conflicts of interest regarding the publication of this article.

## AUTHORS' CONTRIBUTIONS

Zhixiong Zhong, Heming Wu, and Wei Zhong designed the study. Zhixiong Zhong and Heming Wu performed the experiments. Wei Zhong and Qifeng Zhang recruited subjects and collected clinical data. Zhikang Yu helped to analyze the data. Heming Wu prepared the article. All authors were responsible for critical revisions, and all authors read and approved the final version of this work.

## ETHICAL APPROVAL

The study was conducted on the basis of the Declaration of Helsinki and was supported by the Ethics Committee of the Meizhou People's Hospital (Huangtang Hospital).
